# The Impact of Diet on Lipoprotein(a) Levels

**DOI:** 10.3390/life14111403

**Published:** 2024-10-31

**Authors:** Michał Stojko, Aleksandra Spychał, Kamil Nikel, Rafał Kołodziej, Jolanta Zalejska-Fiolka

**Affiliations:** 1 Scientific Society of the Department and Chair of Biochemistry, Faculty of Medical Sciences in Zabrze, Medical University of Silesia in Katowice, 40-055 Katowice, Poland; s81359@365.sum.edu.pl (A.S.); s79826@365.sum.edu.pl (K.N.); 2 Students Scientific Association, Department of Immunology, Faculty of Medical Science, University of Rzeszów, 35001 Rzeszów, Poland; rk112690@stud.ur.edu.pl; 3 Department and Chair of Biochemistry, Faculty of Medical Sciences in Zabrze, Medical University of Silesia in Katowice, 40-055 Katowice, Poland; jzalejskafiolka@sum.edu.pl

**Keywords:** lipoprotein(a), diet, fats, lipids

## Abstract

Background: Lipoprotein(a) [Lp(a)] is recognized as an independent risk factor for cardiovascular diseases; however, the impact of fat-based diets on its levels remains unclear. Objective: This study aims to assess and analyze current evidence on the impact of various types of fat-based diets on Lp(a) levels. Material and Methods: A comprehensive search of the PubMed database was conducted on 9 July 2024, focusing on clinical and randomized trials published since 2000. Out of 697 identified studies, 33 met the inclusion criteria and were selected for analysis. Results: The findings suggest that modifications in fat-based diets, particularly concerning the type and amount of consumed fats and fatty acids, can significantly influence plasma Lp(a) levels. Diets rich in unsaturated fats, including polyunsaturated and monounsaturated fatty acids, were associated with more favorable effects in lowering Lp(a) levels. In contrast, diets high in saturated fats were linked to elevated Lp(a) levels. However, these conclusions were not consistent across all studies considered. Conclusions: This work highlights the importance of a personalized dietary approach, considering both genetic predispositions and dietary habits. While diet alone may not drastically alter Lp(a) levels due to their strong genetic determination, a comprehensive strategy involving a healthy diet rich in unsaturated fats, regular physical activity, and effective weight management is recommended to reduce the risk of cardiovascular diseases. Further research is needed to clarify the mechanisms through which different fats affect Lp(a) and to develop targeted dietary recommendations.

## 1. Introduction

Lipoprotein(a) [Lp(a)] is a complex present in blood plasma, consisting of one LDL particle containing apoB-100 and a large, polymorphic glycoprotein apo(a). The LPA gene responsible for the production of Lp(a) is primarily transcribed in the liver [1]. Plasma Lp(a) concentrations exhibit significant individual variability and are inherited. Discovered by Kåre Berg in 1963, Lp(a) has attracted interest due to its association with atherosclerotic diseases, particularly coronary heart disease (CHD), despite its unclear physiological role [2].

One of the challenges in quantifying Lp(a) is the size polymorphism of apo(a). It is hypothesized that differences in apo(a) size may affect test results, depending on the assay used and its antibody specificity [3]. Despite diagnostic challenges, the fact remains that high Lp(a) levels impact cardiovascular risk (CVR). Epidemiological studies suggest that approximately 20% of the European population has high Lp(a) levels, which is also more common in individuals with familial hypercholesterolemia, further increasing CVR. Effective early treatment to reduce Lp(a) levels is crucial in lowering this risk [4].

Lp(a) also inhibits fibrinolysis, linking cholesterol transport with the coagulation system [5]. Studies also indicate the potential of Lp(a) as an acute-phase protein and its ability to carry oxidized phospholipids, which may contribute to atherosclerosis development [6,7]. This includes its mediation in monocyte adhesion and migration through interaction with β2-integrin Mac-1, which is key in the inflammatory process and plaque formation [8].

To date, several therapeutic options exist to lower Lp(a) levels in the blood [9]. Statins lower LDL cholesterol levels, but their effect on Lp(a) is variable. For example, ezetimibe reduces Lp(a) by about 7%, PCSK9 inhibitors by 23–25%, and mipomersen by 26.4% [10,11]. Other methods, such as microsomal triglyceride transfer protein inhibitors and cholesterol ester transfer protein inhibitors, niacin, and thyroid hormone mimetics, can reduce Lp(a) by 20–30% [12,13,14]. Aspirin may also lower Lp(a), particularly in individuals with high baseline levels [15]. Lipoprotein apheresis is the most effective, reducing Lp(a) by 60–90% [16]. However, therapy choice remains dependent on the individual characteristics of the patient [9].

For this reason, a detailed literature review was conducted to evaluate the impact of the least invasive method of changing plasma Lp(a) levels, namely dietary modification. This work compares how dietary changes may influence Lp(a) levels, which could be crucial in preventing cardiovascular events in individuals with high levels of this lipoprotein.

Dietary interventions can influence Lp(a) levels through several biological pathways. One key pathway is via alterations in hepatic lipid metabolism, as the liver is the primary site of Lp(a) production. Diets rich in unsaturated fats, particularly omega-3 fatty acids, may reduce Lp(a) synthesis by modulating transcription factors involved in lipid homeostasis, such as peroxisome proliferator-activated receptors (PPARs) and sterol regulatory element-binding proteins (SREBPs). Furthermore, diets high in fiber, especially soluble fiber, may improve bile acid excretion, leading to a compensatory increase in hepatic LDL receptor activity, which can lower circulating Lp(a) levels indirectly. Certain dietary patterns, like those rich in polyphenols, have been shown to reduce oxidative stress and inflammation, both of which are associated with increased Lp(a) levels. In contrast, diets high in saturated fats and trans fats can elevate Lp(a) by upregulating inflammatory pathways and LDL particle production, potentially increasing the hepatic secretion of Lp(a). Additionally, certain amino acids, like lysine, have been proposed to interfere with the binding of Lp(a) to fibrinogen, reducing its pro-atherogenic effects. Finally, alcohol consumption in moderate amounts has been shown to lower Lp(a) levels, possibly through enhanced clearance mechanisms, although the exact mechanisms remain under investigation.

## 2. Materials and Methods

Search Methods: The “PubMed” database was searched. As of 9 July 2024, entering the keywords “lipoprotein (a), diet” into the PubMed search engine yielded 697 results, of which 128 remained after narrowing down to research studies. Ultimately, 33 studies were included in the review. The time criteria included studies published from 2000 to the date of the search. There were no restrictions on the publication status regarding entries in the registry.

Objectives: The objective of this study was to analyze and evaluate current evidence on the effects of various fat-based diets on Lp(a) levels.

Selection Criteria: This review included clinical and randomized controlled trials involving humans. Only English-language studies were considered. Studies that ambiguously demonstrated the effect of diet on Lp(a), lacked lipid fractionation, including the separation of Lp(a), or involved changes in pharmacological treatment during the study period were excluded.

**Figure 1:**
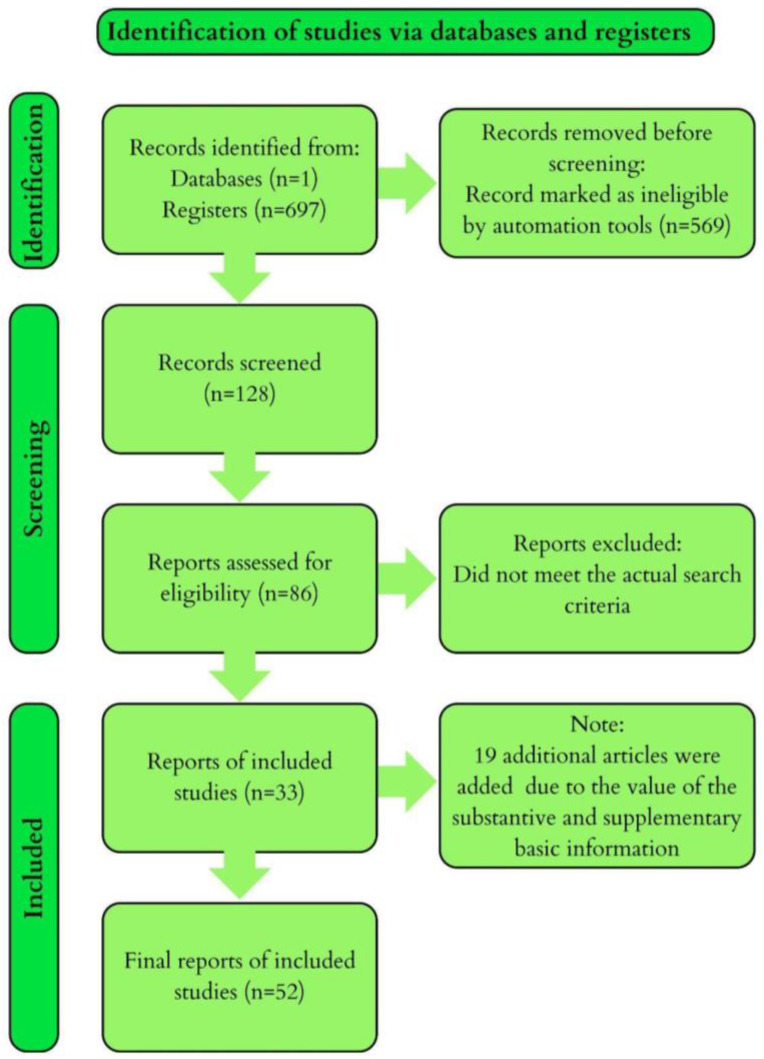
The results of our research visualized in the PRISMA diagram.

Main Results: The review included 33 records of varying quality and sample size. The selected studies are research papers that are statistically significant and directly related to the topic of the impact of diet on Lp(a) levels. An additional 7 studies were included to supplement the review with information provided in the introduction. The results are shown in the PRISMA diagram (Figure 1).

## 3. Results

The results we collected are presented in the form of a table (Table 1) with detailed descriptions of all studies included in the review.

## 4. Discussion

Research on the impact of different types of diets on Lp(a) levels has shown effects dependent on the type of fat. In the context of dietary interventions that may affect Lp(a) levels, it is crucial to consider both the amount and type of fats consumed. Literature examples indicate varying effects of different types of fats. For instance, H.G. Prawo et al., using a diet with reduced total fat and SFA for 12 weeks, observed an increase in Lp(a) levels while simultaneously reducing LDL-C. Similarly, in the study by M.P. St-Onge et al., involving 45 participants over three phases lasting 25 days each, a diet rich in PUFA led to an increase in Lp(a), while a low-fat diet decreased Lp(a) levels. On the other hand, U. Hoppu et al. observed a gradual increase in Lp(a) from the beginning of the study to the fourth year in 127 mothers and their children with reduced SFA intake and increased MUFA and PUFA intake. It is possible that the increase in Lp(a) in this research may be related to other long-term changes in the body that are not directly linked to diet [17,45,47]. 

Delgado-Alarcón et al. compared the impact of three different breakfasts on Lp(a) levels over a month, finding that meals rich in PUFA and MUFA led to significant reductions of Lp(a). In contrast, the 6-week study by A.M. Tindall et al., with similar diets, did not show changes in Lp(a) levels, although the sample size was about half as large (34 people). Similarly, W. Stonehouse et al. found no change in Lp(a) in a study with a similar number of participants and diets. Likewise, S. Gulati et al., in a 24-week period enriched with MUFA and PUFA, did not achieve statistically significant changes in Lp(a). The studies by R. Loganathan et al., S. Vega-Lopez et al., and A.H. Lichtenstein et al. also did not observe significant differences in Lp(a) between different diets enriched with unsaturated fatty acids [18,19,20,24,25,26,27].

The study by S. Vega-Lopez et al. with 30 participants tested a diet enriched with partially hydrogenated soybean oil, which led to a decrease in Lp(a). Soybean oil is primarily a source of PUFA but also contains MUFA and saturated fats [49]. In a larger group (124 people), C. Bamberger et al.’s 8-week study found no significant impact of walnut-enriched diets on Lp(a) levels. Walnuts are rich in various types of fats, including MUFA and PUFA and a small amount of saturated fats [50], making them a focus of interest as a dietary addition. Similarly, pecans, which have a higher MUFA content compared to PUFA, were included in the diet in the study by S. Jaranam et al. and led to a decrease in Lp(a). A decrease in Lp(a) was also observed in the study by D.J.A. Jenkins et al. with almond supplementation, which is mainly a source of monounsaturated fats, while the same dietary addition in J.F. Ruisinger et al.’s study did not result in significant differences in Lp(a) [21,31,32,33].

A small group of 20 participants in D. Iggman’s study examined the effect of canola oil, a source of unsaturated fatty acids, on lipid profiles compared to a dairy-based diet rich in saturated fatty acids. The canola oil diet increased Lp(a) levels. In a study with 162 participants, B. Vessby et al. found that consuming a diet rich in MUFA increased Lp(a) by 12%, although trans fats were at the same level across all tested diets. Conversely, D. Zambón et al. demonstrated a reduction of Lp(a) with a diet supplemented with monounsaturated fats over 6 weeks, with significant decreases observed only in men. Supplementation with flaxseed oil significantly reduced Lp(a) gene expression in peripheral blood mononuclear cells compared to the placebo group in the study by A.A. Hashemzadeh et al. Flaxseed oil is a rich source of fats, particularly PUFA [21,23,28,29].

J.M. Gaullier et al. studied the impact of linoleic acid, free fatty acids, and triglycerides at different concentrations in diets with 180 overweight adults, observing a significant increase in Lp(a). The following year, the same authors published a study where 134 participants from the first study received 3.4 g of linoleic acid daily for another year, which significantly increased Lp(a) after 24 months [34].

In the study by Gebauer S.K. et al. with 106 healthy adults over 24 days, diets with vascenic acid, industrially produced trans fatty acids, and conjugated linoleic acid cis-9, trans-11 (c9,t11-CLA) showed a change only with vascenic acid (increasing Lp(a)). In the study by S.H. Vermunt et al., neither a diet high in trans isomers of alpha-linolenic acid nor a low one showed changes in Lp(a). Similarly, in M. Pfeuffer et al.’s study, including safflower oil, showed no changes. The study by M. Ohman et al. on omega-3 fatty acid supplementation also showed no changes in Lp(a). Participants in P.T. Voon et al.’s study consumed three different diets over 5 weeks, where fats constituted two-thirds of 30% of calories from fat, and only the diet containing coconut oil reduced postprandial Lp(a). This is because long-chain SFA increases postprandial Lp(a), but coconut oil contains shorter-chain fatty acids that lower postprandial Lp(a) [41,42,43,46,48].

The study by T. Tholstrup et al. with 16 healthy men consuming meals with 1 g of fat (mainly saturated) per kg of body weight after a 12 h fast, including stearic and palmitic acids, led to elevated Lp(a) levels. In contrast, the study by C. Seidel et al. with 15 women and 16 men showed that a diet with reduced saturated fatty acids over 13 weeks resulted in the lowest Lp(a) levels compared to standard diets.

It is also worth noting the variability in the results obtained by researchers. When analyzing the available data, it should be emphasized that some studies were based on smaller sample sizes or were of variable quality, which could have affected the reliability and clarity of the results [35,36].

Some studies focused on phytosterols, natural compounds present in various plant parts. The average person consumes 100–400 mg of phytosterols daily, mainly from vegetable oils, bread, cereals, nuts, and vegetables. Due to their cholesterol-like structure, they compete with cholesterol in the intestine, reducing its absorption and lowering LDL-C levels in plasma [51,52]. However, studies by S.S. AbuMweis et al., Y.M. Chan et al., A. Garoufi et al., and M.B. Madsen did not show an impact on Lp(a) levels with phytosterol-enriched diets. In contrast, J.M.M. Fito et al. found a significant reduction of Lp(a) after 6 months of a plant-rich diet among 930 individuals with high CVD risk [37,38,39,44].

Research has demonstrated that the type of fat in the diet can influence Lp(a) levels. Diets high in SFA generally increase Lp(a) levels, whereas reducing SFA in the diet sometimes leads to an increase in Lp(a) levels but more commonly results in a reduction of LDL-C without affecting Lp(a). Diets rich in MUFA and PUFA can have varying effects. Increasing MUFA in the diet may either increase or decrease Lp(a) levels, depending on whether it is the sole dietary supplement and the percentage of fats in the diet as well as the duration of the intervention. Similarly, PUFA can affect Lp(a) in different ways. Some studies show an increase in Lp(a) with PUFA-rich diets, while others show a decrease. Reducing SFA intake and increasing MUFA and PUFA can have a beneficial impact on the overall lipid profile, though the effect on Lp(a) may vary. The diversity in the effects of SFA, MUFA, and PUFA on Lp(a) levels results from genetics, metabolism, diet composition, and the specific characteristics of each fat. Each of these variables can influence study outcomes and the body’s response. Lifestyle and physical activity can also significantly influence Lp(a) levels. Regular physical activity contributes to improving the lipid profile, which can lead to a reduction of Lp(a) levels by decreasing inflammation and enhancing lipid metabolism. Conversely, lifestyle factors such as smoking, excessive alcohol consumption, and obesity can raise Lp(a) levels and increase the risk of cardiovascular diseases. This phenomenon is associated with hormonal and metabolic changes, as well as the development of chronic inflammation, which can stimulate Lp(a) production and lead to disturbances in lipid metabolism. An important topic to address is the adaptation of the diet to demographic and genetic profiles. Dietary interventions must be tailored to genetic and demographic profiles, as responses to dietary changes vary between individuals. For example, people with familial hypercholesterolemia or certain ethnic groups may metabolize fats differently, requiring a more targeted approach. Personalized nutrition, which takes into account genotype and ethnic background, allows for better control of Lp(a) levels and other lipids, potentially reducing the risk of cardiovascular diseases more effectively.

Coconut oil: containing short-chain fatty acids, coconut oil may lower postprandial Lp(a). Nuts and seeds: dietary supplementation with nuts, such as almonds, pecans, or walnuts, may contribute to lowering Lp(a) levels. Flaxseed oil: flaxseed oil has been shown to reduce Lp(a) gene expression, which could be beneficial for individuals with high levels of this lipoprotein. Limiting trans fats: trans fats, such as vascenic acid, increase Lp(a) levels, so their elimination from the diet is beneficial. The impact of dietary fats on lipid levels is variable. Generally, increasing dietary cholesterol raises LDL-C levels, but individual responses can differ. Current dietary guidelines recommend consuming a high amount of vegetables, fruits, legumes, nuts, whole grains, and fish, replacing SFA with MUFA and PUFA, reducing cholesterol intake, avoiding processed meats, refined carbohydrates, and sugary drinks, and eliminating trans fats.

## 5. Summary

Elevated Lp(a) levels are a genetically regulated, independent CVD risk factor. However, variability in Lp(a) levels among individuals and population groups also suggests a role for non-genetic factors. Diets with lower saturated fat content have a moderate impact on Lp(a) levels, usually increasing them, in contrast to LDL cholesterol, which decreases. Diets rich in MUFA and PUFA can either decrease or increase Lp(a) depending on the diet duration, fat content percentage, and supplements used.

Research shows that the effect of diet on Lp(a) levels is variable; however, a common trend is the claim that adopting a diet rich in vegetables, fruits, legumes, nuts, whole grains, and fish, replacing SFA with MUFA and PUFA, reducing cholesterol intake, and avoiding trans fats may benefit the overall lipid profile and cardiovascular health. Nevertheless, this subject requires further study to draw firm conclusions and establish recommendations for controlling Lp(a) levels.

## Figures and Tables

**Table 1:** Publications Investigating the Impact of Diet on Lp(a) Levels.

Authors and Year of Publication	Study Population	Dietary Interventions	Conclusions
H.G. Prawo et al., 2023, [17]	A total of 166 African American individuals aged 18–65 years, without comorbidities.	Diet 1—similar to the average American diet, Diet 2—with lower levels of total fat (25% of energy intake) and saturated fats (6% of energy intake). Carbohydrates were mainly derived from fruits and vegetables. Duration: 12 weeks.	Reducing saturated fatty acid intake significantly increased Lp(a) levels while simultaneously decreasing LDL-C.
J.M. Delgado-Alarcón et al., 2020, [18]	A total of 66 women at risk of cardiovascular disease.	Participants were randomly assigned to three groups, each consuming the following for breakfast for 30 days: Group A: breakfast rich in polyunsaturated fatty acids (PUFA); Group B: breakfast rich in saturated fatty acids (SFA); Group C: breakfast rich in monounsaturated fatty acids (MUFA)	Breakfast rich in PUFA or MUFA reduced Lp(a) levels.
A.M. Tindall et al., 2020, [19]	A total of 34 individuals at risk of cardiovascular disease, including 62% men, average age 44 ± 10 years, with BMI 30.1 ± 4.9 kg/m^2^.	Participants underwent three different diets in random order, each lasting 6 weeks: nut diet: 57–99 g/d walnuts, 7% SFA, 16% PUFA, 9% MUFA. Fat-adjusted nut diet: 7% SFA, 16% PUFA, 9% MUFA. Oleic acid diet replaces α-linolenic acid diet: 7% SFA, 14% PUFA, 12% MUFA.	Lp(a) did not change with any of the diets.
W. Stonehouse et al., 2019, [20]	A total of 38 healthy participants aged 20–40 years.	Participants underwent three different diets in random order, each lasting 4 weeks: palm olein: rich in SFA with unsaturated fatty acids cocoa butter: rich in SFA with unsaturated fatty acids olive oil: unsaturated fatty acids	No significant differences between diets concerning Lp(a).
A.A. Hashemzadeh et al., 2017, [21]	A total of 60 patients with overweight, type 2 diabetes, and coronary artery disease.	Participants were randomly assigned to two groups. The study group received 1000 mg of n−3 fatty acids from flaxseed oil, containing 400 mg α-linolenic acid (ALA, 18:3n−3), twice daily for 12 weeks. The control group received a placebo.	Flaxseed oil supplementation significantly reduced Lp(a) gene expression in peripheral blood mononuclear cells compared to the placebo group.
C. Bamberger et al., 2017, [22]	A total of 194 healthy individuals, average age 63 ± 7 years, with BMI of 25.1 ± 4.0 kg/m^2^.	Participants underwent two 8-week dietary periods: (1) diet enriched with 43 g of walnuts daily (saturated fats) and reduced fat and carbohydrate intake; (2) diet without walnuts.	Walnut consumption had no significant impact on Lp(a) levels.
D. Iggman et al., 2011, [23]	A total of 20 individuals with hypercholesterolemia.	The study assessed the impact of canola oil, a source of unsaturated fatty acids, on lipid profiles compared to a dairy-based diet rich in saturated fatty acids. Participants followed two different diets for two 3-week periods: saturated fat diet from dairy products (DF); diet with fat based on canola oil (RO). Both diets were isocaloric and differed only in fat composition.	RO diet slightly increased Lp(a) levels by 6% (P = 0.05).
S. Gulati et al., 2017, [24]	A total of 50 individuals aged 25–70 with type 2 diabetes, taking stable doses of metformin, with HbA1c < 9% and LDL-c ≥ 100 mg/dl.	Patients underwent a 3-week control diet and exercise period, followed by a 24-week period consuming raw almonds (MUFA and PUFA) making up 20% of daily energy intake, replacing fats and a portion of carbohydrates.	Changes in Lp(a) levels did not reach statistical significance.
R. Loganathan et al., 2022, [25]	A total of 40 healthy individuals aged 20–50 years.	Participants were randomly assigned to one of three groups, each receiving baked goods enriched with unsaturated fatty acids (brownies for breakfast and cookies for a snack) prepared with: (1) palm olein, (2) cocoa butter, (3) extra virgin olive oil.	No significant differences in Lp(a) between the different diets.
S. Vega-Lopez et al., 2006, [26]	A total of 15 volunteers aged ≥50 years with LDL cholesterol ≥130 mg/dL.	Participants consumed food based on one of four diets for 35 days per phase. The diets differed by type of fat: partially hydrogenated soybean oil, soybean oil, palm oil, or canola oil, with two-thirds of the fat coming from the respective oil, comprising 20% of the diet’s energy.	No effect of the studied fats on Lp(a) in plasma.
A.H. Lichtenstein et al., 2006, [27]	A total of 30 individuals (16 women and 14 men) over 50 years old with moderate (LDL cholesterol > 130 mg/dl) hypercholesterolemia.	Participants consumed five different diets, each for 35 days in random order. The diets contained the same foods and provided 30% of energy from fat, with two-thirds from one of the following oils: soybean oil (SO), low SFA soybean oil (LoSFA-SO), high oleic soybean oil (HiOleic-SO), low ALA soybean oil (LoALA-SO), partially hydrogenated soybean oil (Hydrog-SO).	Consumption of the studied oils had no significant impact on Lp(a) levels in blood.
D. Zambón et al., 2000, [28]	A total of 55 individuals, average age 56 years with polygenic hypercholesterolemia.	Participants followed two different diets for 6 weeks each: Mediterranean diet and a diet with a similar energy and fat content, with walnuts replacing about 35% of energy from monounsaturated fats.	Reduction of Lp(a) by 6.2%, with a significant decrease observed only in men.
B. Vessby et al., 2001, [29]	A total of 162 healthy, randomly selected individuals.	The main goal was to check whether a diet rich in MUFA affects insulin sensitivity. Participants were divided into two groups, receiving isocaloric diets rich in saturated fats and monounsaturated fats. Additionally, participants were randomly assigned to subgroups supplementing with fish oil (3.6 g n−3 fatty acids daily) or placebo.	Consumption of a diet rich in MUFA increased Lp(a) by 12%.
S. Vega-Lopez et al., 2009, [30]	A total of 30 postmenopausal women aged ≥50 years with LDL cholesterol ≥120 mg/dL.	Participants consumed diets enriched with two different fats for two periods of 35 days each: corn oil (control), partially hydrogenated soybean oil. Each diet included two-thirds of fat from the respective oil. All meals and drinks were provided to maintain stable body weight.	Corn oil diet, compared to partially hydrogenated soybean oil diet, lowered Lp(a) levels.
S. Jaranam et al., 2001, [31]	A total of 23 healthy participants, average age 38 years.	In a double-blind, randomized controlled trial, the effect of a diet rich in pecans (unsaturated fats) on lipid profiles was compared. Participants were randomly assigned to a diet containing 28.3% energy from fat or a diet enriched with pecans. Participants replaced 20% of calories from Step I diet with pecans.	A diet enriched with pecans led to a significant decrease in Lp(a) levels.
D.J.A. Jenkins et al., 2002 [32]	27 men and women with hypercholesterolemia	In a randomized crossover trial, the effect of consuming almonds (unsaturated fat) as a snack was compared with low-fat (<5% energy) whole-grain muffins as a control group, participants consumed an isocaloric diet for 1 month	Lp(a) concentration decreased significantly (7.8 ± 3.5%, P = 0.034)
J.F. Ruisinger et al., 2015 [33]	48 people who received a stable dose of a statin for years.	Subjects were randomly assigned to two groups: Almond (unsaturated fat) group (n = 22): addition of 100 g almonds per day to the diet and dietary advice consistent with the Third Assessment Report of the Adult Treatment Panel on Lifestyle Changes. No almond group (n = 26): dietary advice only consistent with the Third Assessment Report of the Adult Treatment Panel on Lifestyle Changes.	No significant differences were observed in Lp(a)
J.M. Gaullier et al., 2004 [34]	180 healthy overweight adults (BMI 25–30 kg/m^2^)	Participants were randomly assigned to one of three groups for 12 months: linoleic acid (CLA)-free fatty acid (FFA) CLA-triacylglycerol Placebo (olive oil)	A statistically significant increase in Lp(a) was observed in the study groups.
J.M. Gaullier et al., 2005 [34]	The study included 134 of 157 participants who completed the initial 12-month study. All participants were healthy overweight adults.	In this study, all participants received 3.4 g of CLA daily as triglycerides for the next 12 months.	Conjugated linoleic acid supplementation for 24 months significantly increased blood Lp(a) concentration
T. Tholstrup et al., 2004 [35]	16 healthy young men	The aim of the study was to investigate the effect of individual fatty acids on postprandial Lp(a) levels and its relationship with lipemia and tissue plasminogen activator (t-PA). Participants consumed meals containing the tested fats (1 g fat/kg body weight) after a 12-h fast. The tested fats were dominated by (approximately 43% g/kg) stearic (S), palmitic (P), oleic, C18:1 trans (T), or linoleic acid. The fats were administered on random days separated by 3-week washout periods.	After consuming meals containing the tested fats, a significant increase in Lp(a) concentration was observed, and the Lp(a) response was different depending on the type of fat. T fat did not change Lp(a) concentration during the study. No relationship was observed between Lp(a) and t-PA concentrations. S and P saturated fats caused an increase in Lp(a), T fat showed a higher response to triacylglycerols (TAG)
C. Seidel et al., 2004 [36]	31 people (15 women and 16 men), nine of whom had hypercholesterolemia	The aim of the study was to compare the effect of dairy products with modified milk fat (ModFat—reduced content of saturated fatty acids) with regular milk fat (RegFat) and soft margarine (Marg) on the concentration of cholesterol, TAG, Lp(a) in the blood of the subjects. The study lasted 13 weeks.	The lowest Lp(a) concentration was shown during the ModFat treatment period compared to other diets.
S.S. AbuMweis et al., 2006 [37]	30 people with mild to moderate hypercholesterolemia	The study aimed to determine the effects of two novel plant sterol formulations on plasma lipids: plant sterols combined with fatty acids from fish oil or esterified to these fatty acids. Participants consumed the following formulations for 29 days as a single dose with a morning meal	None of the plant sterol preparations significantly changed Lp(a) concentration.
Y.M. Chan et al., 2007 [38]	21 moderately overweight individuals with hypercholesterolemia	Patients consumed three different treatment diets, each for 28 days, with 4-week washout periods between diets, in a randomized crossover design: Diet containing olive oil (OO). Diet containing plant sterols esterified to sunflower oil fatty acids (PS-SO). Diet containing plant sterols esterified to olive oil fatty acids (PS-OO). Each diet contained 30% energy from fat, of which 70% came from olive oil. PS-SO and PS-OO provided 1.7 g of plant sterols per day.	No differences in Lp(a) concentrations were observed between diets. However, Lp(a) concentrations increased after OO and PS-SO diets (P = 0.0050 and 0.0421, respectively).
A. Garoufi et al., 2014 [39]	59 children aged 4.5 to 15.9 years, 25 of whom had an initial LDL-C level of 3.4 mmol/l (130 mg/dl) or higher and 34 had lower	Children with hypercholesterolemia received a yogurt drink enriched with 2 g of plant sterols daily for 6–12 months as an addition to their diet. After this period, participants’ lipid profiles were reassessed.	Lp(a) concentration remained unchanged
M.B. Madsen, 2007 [40]	6 people with mild hypercholesterolemia, mean age 50.6 ± 9.8 years	In the run-in period and two intervention periods, each lasting 4 weeks. The study products consisted of 20 g low-fat margarine (35% fat) and 250 ml low-fat milk (0.7% fat), providing a total of 2.3 g plant sterols per day.	Consumption of products enriched with plant sterols had no effect on Lp(a) concentration
Gebauer S.K. et al., 2015 [41]	106 healthy adults, mean age: 47 ± 10.8 years, BMI: 28.5 ± 4.0 kg/m^2^, LDL cholesterol: 3.24 ± 0.63 mmol/l	The study was a 24-day, double-blind, randomized, crossover feeding trial. Control diet (0.1% mixed trans fatty acid (TFA)) Diet containing ~3% vaccenic acid (VA) Diet containing ~3% industrially produced trans fatty acids (iTFA) Diet containing 1% cis-9, trans-11 conjugated linoleic acid (c9,t11-CLA) Dietary fat content: 34% energy	VA increased Lp(a) concentration compared to the control diet (2–6% change). The other diets did not significantly affect Lp(a) concentration.
S.H. Vermunt et al., 2001 [42]	88 healthy men from three European countries: France, Scotland and The Netherlands.	In the study, participants were put on a diet with experimental oils purified from trans alpha-linolenic acid for 6 weeks. In the next stage (study period), participants were randomly assigned to a diet with a high content of trans alpha-linolenic acid (1410 mg per day) or a low content of these isomers.	No effect of a diet rich in trans-linolenic acid isomers on Lp(a) concentration was observed in the study group compared to a diet low in trans-linolenic acid isomers.
M. Pfeuffer et al., 2011 [43]	85 overweight men (aged 45–68, BMI 25–35 kg/m^2^)	4-week, double-blind study. Participants were randomly assigned to one of four groups: 4.5 g/day of a mixture of conjugated linoleic acid (CLA), 4.5 g/day of safflower oil, 4.5 g/day of heated safflower oil, 4.5 g/day of olive oil (control group).	CLA consumption compared to safflower oil did not change Lp(a) concentration
M. Fito et al., 2014, [44]	930 people at high risk of cardiovascular disease, including 420 men and 510 women	A multicenter, randomized, controlled, parallel-group clinical trial that lasted 6 months. The study patients were randomly assigned to three dietary intervention groups: control group (KT) (changes in current dietary habits), low-fat diet (LD) (reduction of total fat intake to <30% of total calories), and plant-rich diet (PD) (reduction of total fat intake to <30% of total calories and increased consumption of plants and dietary fiber)	Lp(a) concentration was significantly reduced in the PD group (mean decrease of 15%), compared to the KT group (no significant change) and the LD group (no significant change).
M.P. St-Onge et al., 2009 [45]	45 study participants. Adult men and women aged 19–65 years, with LDL concentration in the range of 3.37–4.66 mmol/L	The nutritional study was divided into three phases, each lasting 25 days. Participants consumed three different diets that differed in the type of snacks: Low-fat diet (30.8% energy), Moderate-fat diet with saturated fat (37.9% energy from fat, including 11.4% energy from saturated fat), Moderate-fat diet with polyunsaturated fat (36.3% energy from fat, including 9.7% energy from polyunsaturated fat)	The high polyunsaturated fat diet increased Lp(a) in all groups. The low-fat diet decreased Lp(a) in all groups. Participants with low and intermediate baseline CRP had greater decreases in Lp(a) than those with high baseline CRP with the low-fat diet.
M. Ohman et al., 2008 [46]	19 healthy volunteers	The study aimed to evaluate the effect of omega-3 enriched eggs on the lipid profile of volunteers. Participants consumed one additional egg per day: a standard egg or an egg enriched with omega-3 fatty acids. Each period lasted 1 month.	No significant changes in Lp(a) concentration were observed
U. Hoppu et al., 2013 [47]	256 mothers in the first trimester of pregnancy. The number of mothers and their children participating in the study decreased in the following years. Finally, 127 mothers and their children participated in the study by the end of the four-year follow-up period.	Dietary counseling was provided to mothers during pregnancy and breastfeeding. The infants were monitored with 3-day dietary records. Participants were randomly assigned to three groups: diet/probiotics, diet/placebo, and control/placebo. The probiotics included Lactobacillus rhamnosus GG and Bifidobacterium lactis. The study lasted 4 years. Dietary counseling focused on reducing the intake of saturated fatty acids (SFA) and increasing monounsaturated (MUFA) and polyunsaturated (PUFA)	Lp(a) levels increased from baseline to year 4, with mean values increasing from 22.6 mg/dL to 28.6 mg/dL
P.T. Voon et al., 2011 [48]	45 healthy Malaysian adults (9 men, 36 women)	The study examined the effects of a Malaysian high-protein diet prepared with 3 different fats on CVD risk markers in the blood. in 3 dietary periods, each lasting 5 weeks. Participants consumed three different diets in which fats accounted for two-thirds of the 30% of calories from fat: a diet containing palm olein (PO) rich in palmitate (palmitic acid, 16:0), a diet containing coconut oil (CO) rich in lauric acid (12:0) and myristic acid (14:0), and a diet containing olive oil (OO) rich in oleic acid (18:1).	The CO diet reduced postprandial Lp(a) concentration (postprandial concentration in the CO diet 1.31 +/− 6 1.11 mmol/L vs 1.42 in PO and 1.41 in OO) in contrast to the PO and OO diets.
